# Prescription Pattern and Off-Label Use of Antipsychotics in a Middle Eastern Population

**DOI:** 10.3389/fphar.2021.753845

**Published:** 2021-11-01

**Authors:** Kholoud Bastaki, Mohammed El Anbari, Suhaila Ghuloum, Puthen Veettil Jithesh

**Affiliations:** ^1^ College of Health and Life Sciences, Hamad Bin Khalifa University, Doha, Qatar; ^2^ Hamad Medical Corporation, Doha, Qatar; ^3^ Sidra Medicine, Doha, Qatar; ^4^ Weill Cornell Medicine, Doha, Qatar

**Keywords:** antipsychotics, prescription pattern, Qatar, off-label, personalized medicine, electronic medical records, adverse drug effects

## Abstract

**Background:** Understanding the prescription pattern of medications in a population can help reveal the potential usage scenarios, including off-label prescriptions, and the need for precision medicine implementation. Therefore, the aim of this study was to assess the prescription pattern and off-label use of antipsychotics in the Qatari population.

**Methods:** We performed a cross-sectional study of Qatari patients who received antipsychotic prescriptions from the major healthcare providers in the country during the 2-year period between June 2018 and May 2020. The number of patients, prescriptions dispensed, and clinical indications were collected and statistical analysis using chi-square test was conducted.

**Results:** Among the 9,349 Qatari patients prescribed with antipsychotics during the study period, the majority were female (57%; *p* < 0.001) and were in the age categories 20–39 and 30–39 years (both 22%; *p* < 0.001). Among the 35,938 antipsychotic prescriptions dispensed, second-generation antipsychotics were the most highly prescribed (59%), specifically, quetiapine (16%) and olanzapine (12%), but the first-generation antipsychotic prochlorperazine (13%) was also highly prescribed. Most of the indications of antipsychotics (69%) were for off-label use such as for controlling chronic diseases, sleeping disorders, benign paroxysmal positional vertigo and irritable bowel syndrome.

**Conclusion:** Non-mental health and off-label prescriptions of several antipsychotics were observed. Integration of this data with pharmacogenomic and clinical outcome data will help in determining the course of action for implementing personalized and precision medicine in the country and beyond.

## Introduction

Several guidelines exist for the rational prescription of antipsychotics. However, prescribing in real-world settings usually differs from these guideline recommendations (Ramadas et al., 2010). The main indications for the prescription of antipsychotics approved by the U.S. Food and Drug Administration (FDA) are for treating schizophrenia and bipolar disorders as well as for adjunctive treatment of major depressive disorder (MDD) and short-term treatment of generalized anxiety and autism spectrum disorders (ASD). A few first-generation antipsychotics (FGAs) such as chlorpromazine were also approved for non-mental health disorders (non-MHDs) such as severe nausea and vomiting, acute intermittent porphyria and intractable hiccups. The guidelines from the National Institute for Health and Care Excellence (NICE) in the United Kingdom and the American Psychological Association (APA) for borderline personality disorder recommend that using antipsychotics for a short period might be helpful in severe conditions and psychiatric crisis, however, their long-term use is controversial (Kendall et al., 2009; Yadav, 2020). Prescription of antipsychotics for other mental health disorders (MHDs) are also usually not recommended (Marston et al., 2014). Although, these medications could be used for other indications not approved by the guidelines, such as obsessive-compulsive disorder (OCD), irritable bowel syndrome (IBS) and sleeping disorders, they are associated with serious adverse effects (Maglione et al., 2011). These include extrapyramidal syndrome (EPS) with FGAs and metabolic syndrome including weight gain and lipid/glucose abnormalities with the second-generation antipsychotics (SGAs). Therefore, the international guidelines emphasize on the importance of regular monitoring of glucose and lipids profile and body mass index in patients taking antipsychotics for long term (Jibson et al., 2014; Jibson, 2017).

Antipsychotic prescribing has grown beyond their approved indications; however, their benefits, effectiveness and side effects in such off-label uses are unclear. These off-label prescriptions may be for unlicensed indications or may involve deviations from the approved dosage or age group. In a study in the United Kingdom, 65% of psychiatrists stated that they had prescribed a medication off-label within the previous month, with around 50% prescribing for an unlicensed indication (Haw and Stubbs, 2007). In the Middle East, there are few studies that targeted off-label use of medications. One study found that off-label use is mostly in adult and geriatric patients who have diabetes and depression, where the most prescribed medications were antidepressants, antidiabetics, and antipsychotics. It was also noted that the most off-label use was for unlicensed indications, while a lesser extent of prescribing above the recommended dosage range or to patients outside the age range were also observed (Sheblaq et al., 2019). In our recent investigation of the prescribing pattern of antidepressants in the Qatari population, we observed a large number of such off-label prescriptions of several antidepressants (Bastaki et al., 2021). Another study on the pattern of prescription of antipsychotics in Qatar focusing on polypharmacy identified about 30% of the studied received at least two antipsychotics concomitantly (Ouanes et al., 2020).

It is also known that around 30–50% of patients taking antipsychotic treatment do not respond effectively to these medications and develop long-lasting and often severe adverse effects (Pouget et al., 2014; Arranz et al., 2021). Several factors may contribute to such treatment variation between individuals, including clinical, demographic, environmental, and genetic and epigenetics factors (Blazer and Hernandez, 2006; Arranz et al., 2021). In our study of the Qatari population from more than 6,000 whole genome sequences, we identified the distribution of genotypes affecting the metabolism of several antidepressants (Bastaki et al., 2021). Understanding the prescription pattern of antipsychotics is important in prioritizing studies for elucidating the causes of such variation in a population (Jithesh and Scaria, 2017). Systematic collection of prescription data through the use of electronic medical records provides an opportunity to study the pattern and specific diagnostic indications for which such prescribing is made. Therefore, the aim of this study was to explore the prescribing pattern of antipsychotics and to examine the recorded indications for antipsychotic prescriptions in the Qatari population through a systematic retrospective database mining.

## Methods

In this cross-sectional study, we extracted electronic records of Qatari patients receiving antipsychotics from the major healthcare providers in Qatar, Hamad Medical Corporation (HMC) and Primary Healthcare Corporation (PHCC). Inclusion criteria were 1) outpatients of Qatari nationality 2) who were prescribed an antipsychotic 3) during a period of 2 years between June 2018 and May 2020. Records of hospitalized (inpatients) were not included in the study. Mental healthcare in Qatar is almost exclusively provided by HMC, followed by PHCC to a lesser degree. There is only a very limited private practice available for mental health, and hence our sampling was largely reflective of the Qatari patient population in the country. The investigation was done on anonymized electronic medical records (EMR) after obtaining approval from the HMC Medical Research Centre (MRC) and the PHCC Department of Clinical Research.

The number of patients who were prescribed antipsychotics and the number of dispensed prescriptions were assessed. The number of each antipsychotic (FGA or SGA) prescription was extracted from the EMR. To calculate the number of patients, we did not count multiple prescriptions for the same patient. Furthermore, diagnostic indications leading to the prescription of antipsychotics were also extracted from the records, which were present along with each prescription. This data comes from the input of clinicians and not coded. The database of WHO Collaborating Centre for Drug Statistics Methodology was used to obtain the Anatomical Therapeutic Chemical classification (ATC) codes associated with the drug categories.

The chi-square test was used for finding the association between the medications and the age category, as well as the association between the medications and gender. Prescription counts were summarized using balloon graphics, which displayed the magnitude of the count at a given point while crossing the two variables. For all the analysis and plotting, *R* statistical package was employed. For the comparison of on-label and off-label prescriptions, any diagnostic indication for a drug which was not approved by FDA was considered as off-label.

## Results

### Prescription Pattern of Antipsychotics

**FIGURE 1:**
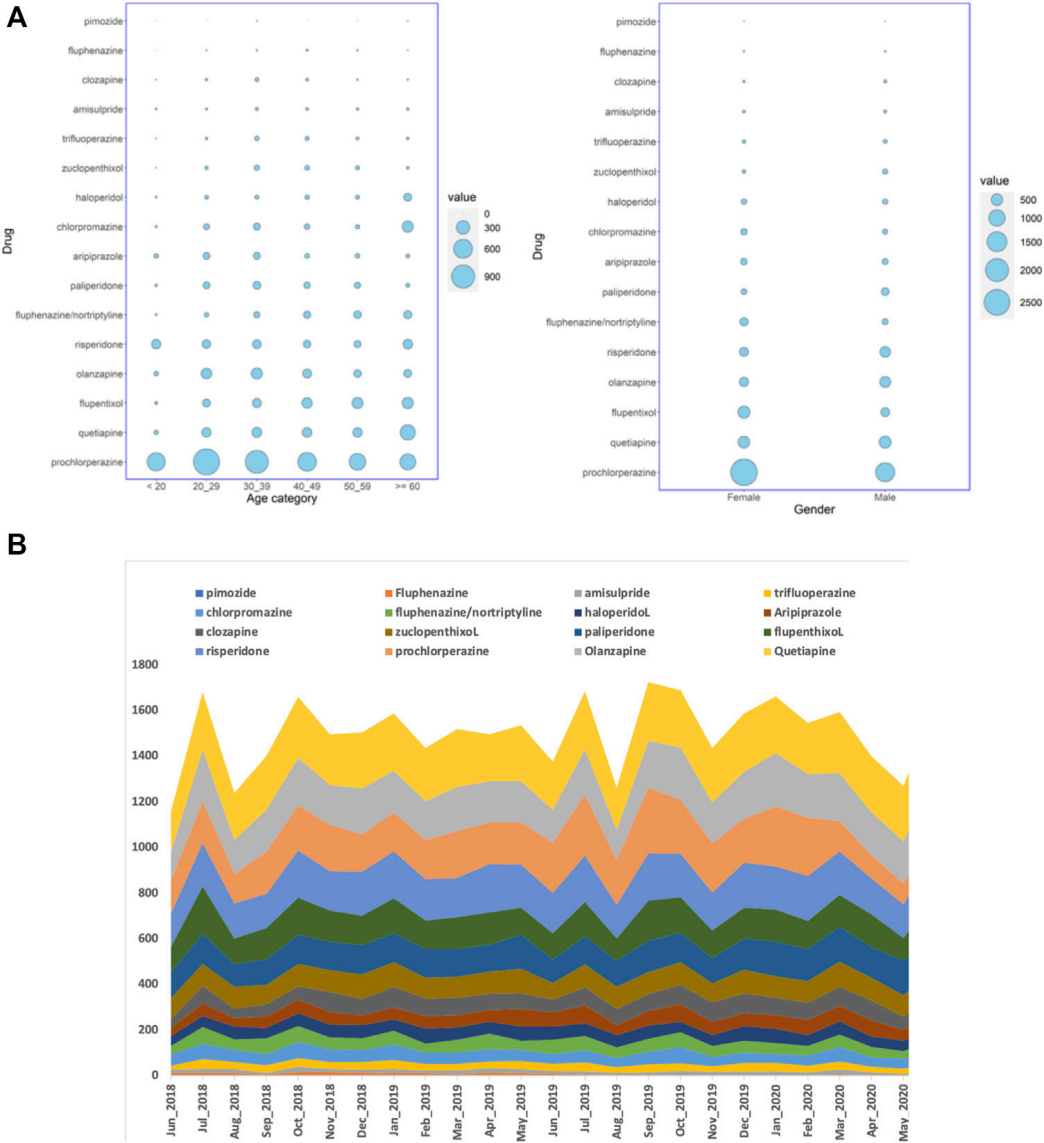
**(A)** Balloon plots showing the distribution of the number of Qatari patients getting antipsychotic prescriptions over a period of 24 months in all the hospitals studied, stratified by either age groups or gender, and **(B)** the number of dispensed antipsychotic prescriptions over the study period of 24 months in all the hospitals. The cumulative number of monthly dispensed prescriptions are shown on the Y-axis and the months on the X-axis.

We studied the electronic medical records of Qataris availing the national healthcare facilities. Though the population of Qatar is around 2.6 million, Qataris account for only about 12% (∼312,000). Among the 9,349 Qatari patients prescribed with antipsychotics during the 2-year study period between June 2018 to May 2020, 5,342 (57%; *p* < 0.001) were female. Statistically significant association of age groups with antipsychotic prescription was also observed, with patients in both 20–29 years (2,039; 22%) and 30–39 years (2,016; 22%) receiving more prescriptions than the other groups (Figure 1A). Both typical or first-generation antipsychotics (FGAs) and atypical or second-generation antipsychotics (SGAs) were prescribed for the population. Main FGAs prescribed were phenothiazines with piperazine structure (ATC code: N05AB) or with aliphatic side chain (N05AA), such as prochlorperazine. Major SGAs prescribed belonged to the class of “diazepines, oxazepines, thiazepines, and oxepines” (N05AH; quetiapine, clozapine, and olanzapine). Among the 9,349 Qatari patients prescribed antipsychotics, prochlorperazine (4,113; 44%) was the most highly prescribed antipsychotic, followed by quetiapine (1,059; 11%) and flupentixol (839; 9%).

When the number of prescriptions were considered instead of the number of patients, during the 24 months, 35,938 antipsychotic prescriptions were dispensed for Qataris at all the hospitals. During the same study period, the number of antipsychotics prescribed for all patients, including both Qataris and non-Qataris, was 82,059, while the total prescription of all type of medications was 24,923,715. SGAs were the most highly prescribed antipsychotics in Qataris (21,030; 59%), with quetiapine (5,669; 16%) at the top, followed by prochlorperazine (4,582; 13%) and olanzapine (4,469; 12%) (Figure 1B).

### Diagnostic Indications for Antipsychotic Prescriptions

**FIGURE 2:**
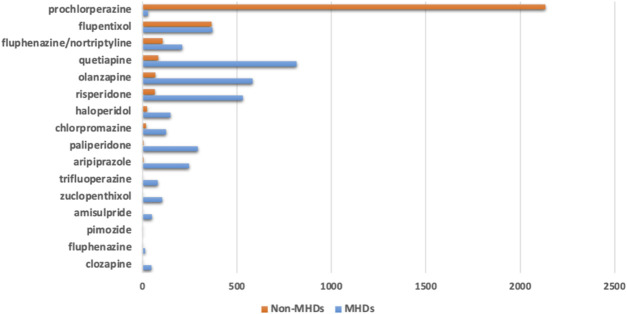
Number of antipsychotic prescriptions based on their diagnostic indications classified into mental health disorders (MHDs) or non-mental health disorders (Non-MHDs).

We identified 3,646 diagnostic indications that may be classified as mental health disorders (MHDs) while 2,884 indications were non-MHDs. Prochlorperazine was prescribed mainly for non-MHDs. Several other antipsychotics, including flupentixol, quetiapine, olanzapine, risperidone, haloperidol, chlorpromazine, and the combination of fluphenazine with nortriptyline were also prescribed for indications which were non-MHDs (Figure 2).

**FIGURE 3:**
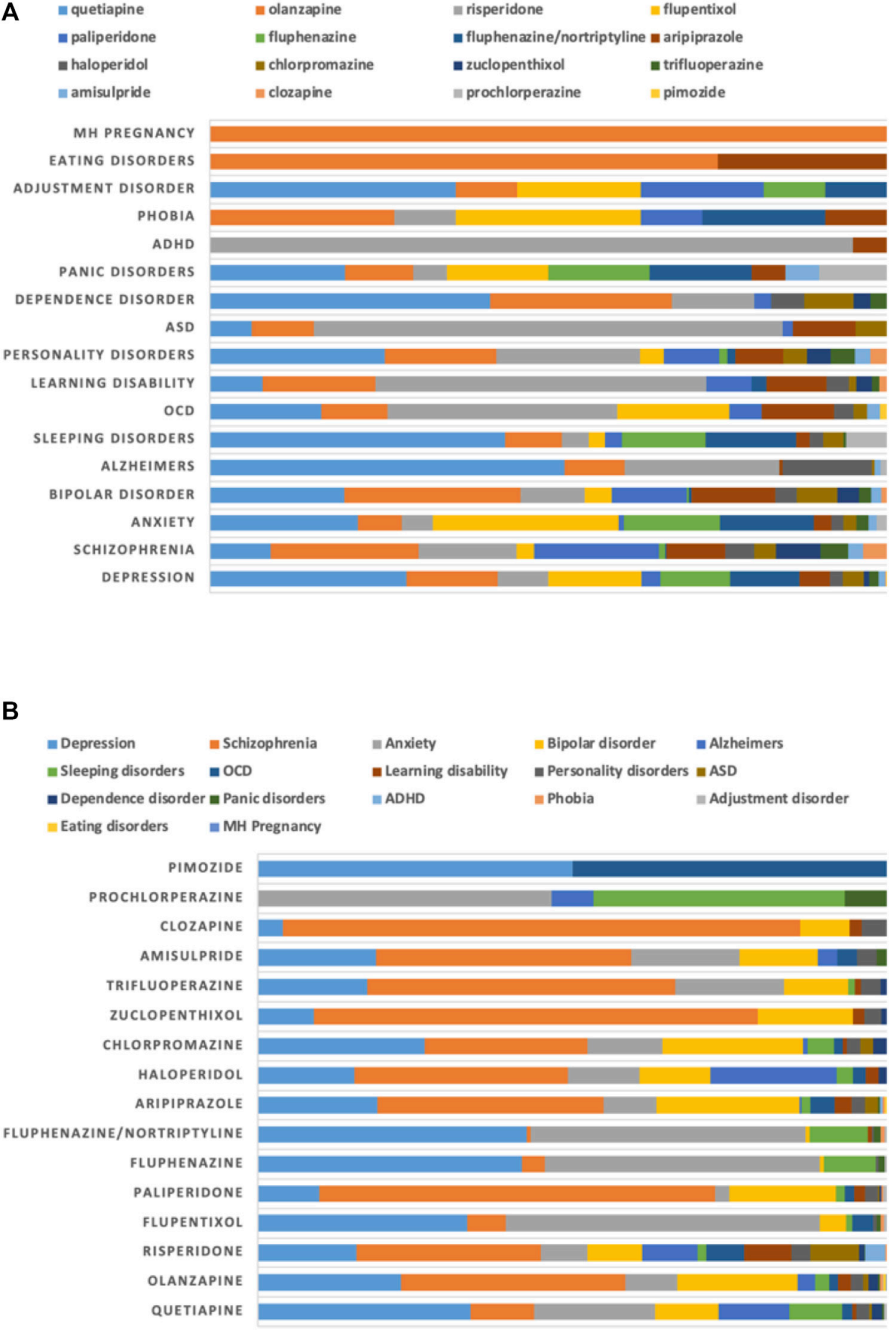
Proportion of use of antipsychotics for mental health disorders (MHDs) stratified by **(A)** diagnoses and **(B)** drugs.

The major diagnostic indications for MHDs were depression (1,226; 26%) and schizophrenia (1,188; 25%), followed by anxiety (928; 20%) and bipolar disorder (534; 11%) (Figure 3A). Quetiapine (356; 29%) was the major antipsychotic prescribed for depression, while flupentixol (169; 14%) and olanzapine (165; 14%) were in the second and third places. Olanzapine (259; 22%), paliperidone (219; 18%) and risperidone (172; 15%) were the major antipsychotics prescribed for schizophrenia (Figure 3B).

**FIGURE 4:**
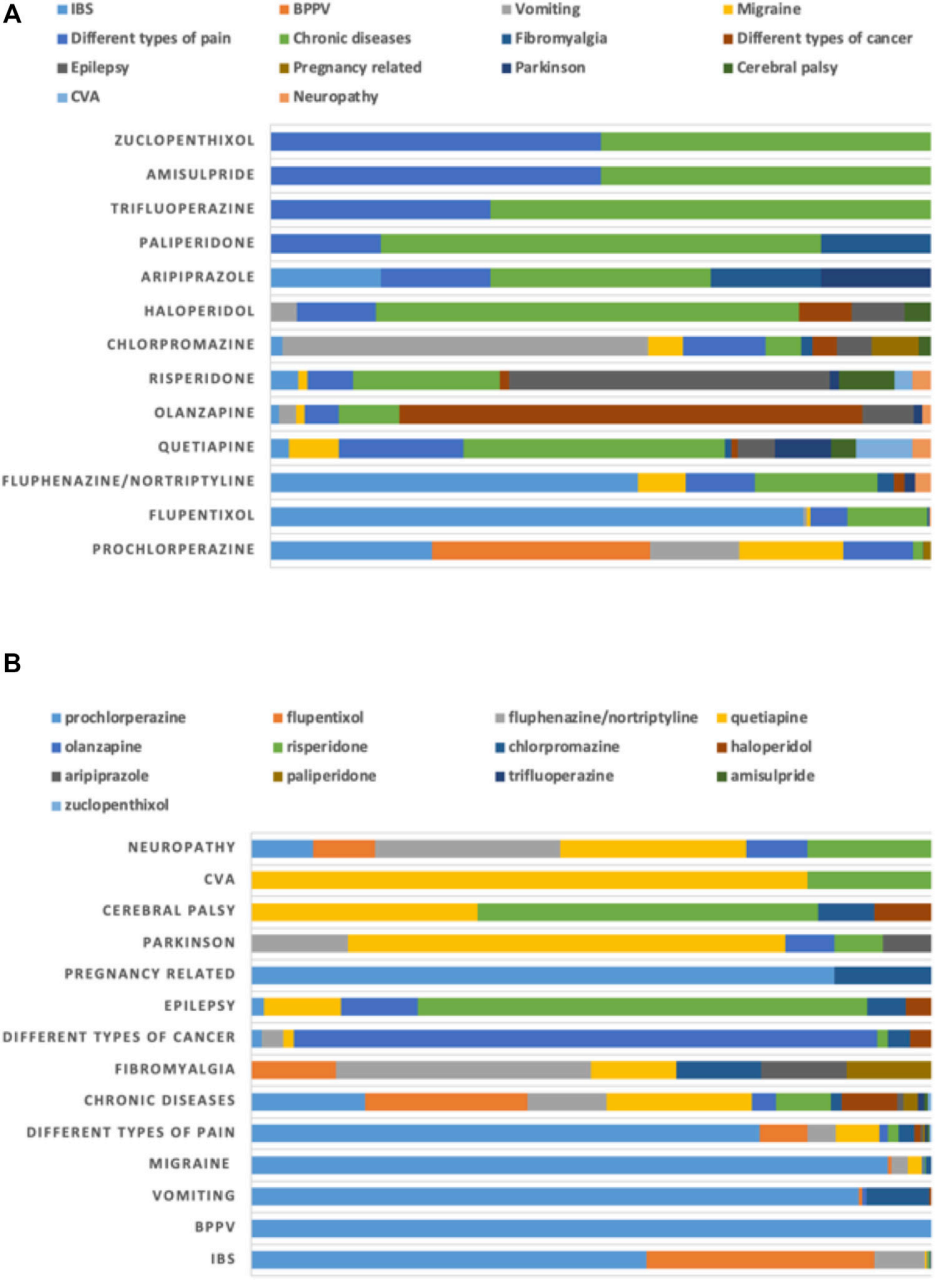
Proportion of use of antipsychotics for non-mental health disorders (non-MHDs) stratified by **(A)** diagnoses and **(B)** drugs.

The major non-mental health disorders for which antipsychotics were prescribed included irritable bowel syndrome (IBS; 935; 30%) and benign paroxysmal positional vertigo (BPPV; 735; 24%), followed by migraine (375; 12%) and vomiting (336; 11%). Prochlorperazine was prescribed heavily for several of these conditions, including BPPV (735; 33%), IBS (543; 24%), migraine (351; 16%) and vomiting (300; 14%). It was also observed that prochlorperazine was the only antipsychotic prescribed for BPPV (Figure 4).

### Off-Label Use of Antipsychotics

Following the latest FDA approval information for each antipsychotic, we identified that 69% of the diagnostic indications were off-label, as these were not approved by FDA. The off-label use in MHDs was 56%, with large number of off-label indications of anxiety (897 out of 928 indications of anxiety) and depression (650 out of 1,226 indications of depression). Some FDA approved use of antipsychotics such as prochlorperazine and trifluoperazine for anxiety, and quetiapine, olanzapine and aripiprazole for depression was also observed. Off-label use of antipsychotics in non-MHD was substantially high (89%), with several indications of IBS and BPPV, as mentioned above, all of which were FDA unlicensed indications. On-label use of prochlorperazine and chlorpromazine for nausea and vomiting was also observed.

### Prescription Pattern of Antipsychotics in the Mental Health Hospital

**FIGURE 5:**
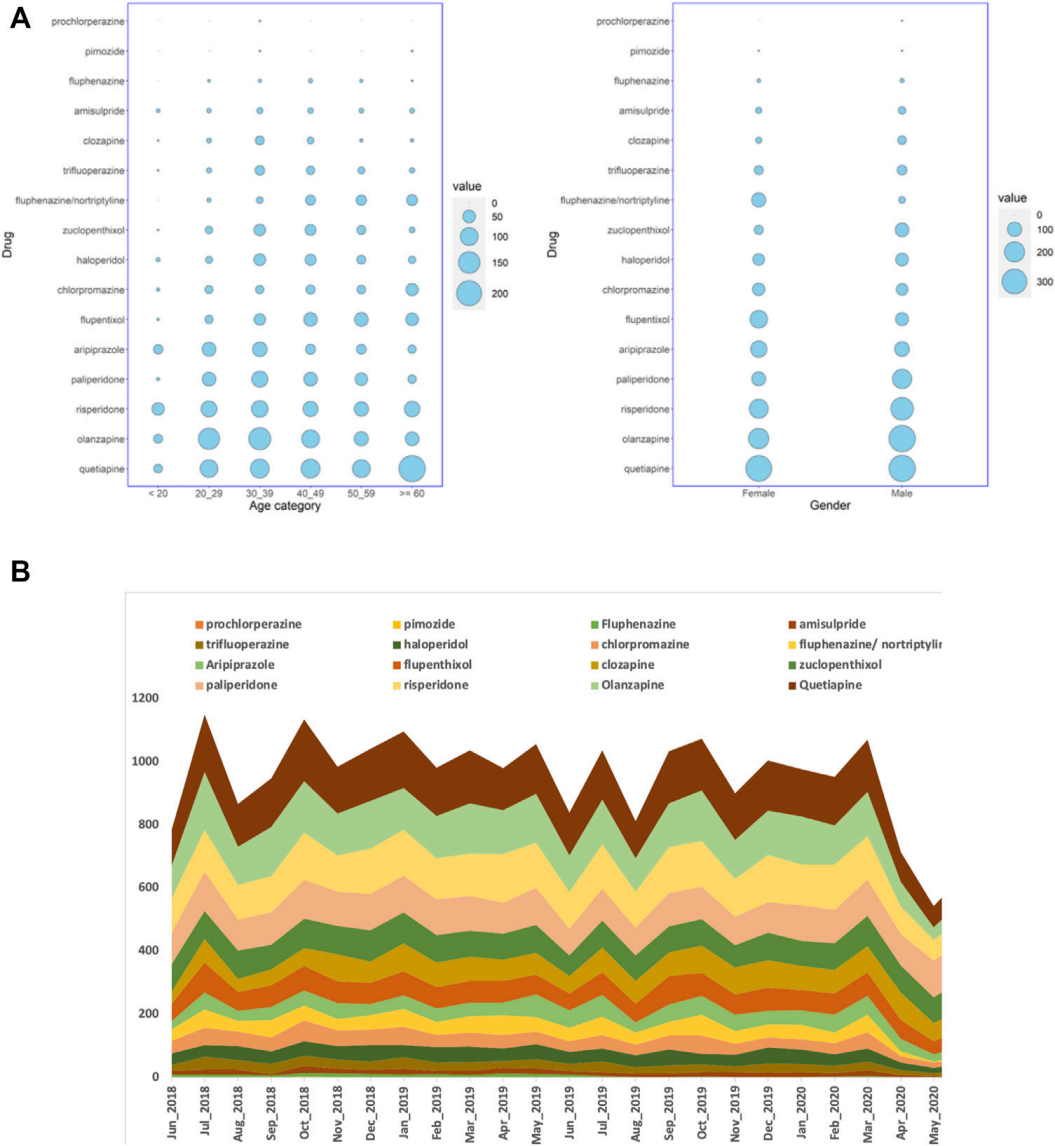
**(A)** Balloon plots showing the distribution of the number of Qatari patients getting antipsychotic prescriptions over the study period of 24 months considering the Mental Health Hospital alone, and stratified by age group or gender, and **(B)** the number of prescriptions of antipsychotics dispensed over a period of 24 months in the Mental Health Hospital alone. The cumulative number of monthly dispensed prescriptions on the Y-axis and the months are shown on the X-axis.

We further studied the prescription pattern in the MHH only as we observed large number of antipsychotic prescriptions for non-MHDs in the general hospital setting. Among the 23,000 antipsychotic prescriptions dispensed at the MHH, there was only one prochlorperazine prescription, which was one of the major drugs prescribed when all the hospitals were considered. In the MHH, quetiapine was still the most highly prescribed antipsychotic (3,561; 16%), followed by olanzapine (3,198; 14%) and risperidone (3,073; 13%). A similar pattern was observed when the number of patients who received antipsychotic prescriptions at the MHH were considered instead of number of dispensed prescriptions (Figure 5).

## Discussion

In this study of the prescription pattern of antipsychotics in the Qatari population from electronic medical records covering the major public healthcare providers in Qatar, we found that antipsychotics were prescribed more for female patients and in the age group of 20–39 years. This result was contradictory with a previous study in Qatar that was targeted at antipsychotic prescribing pattern and polypharmacy, which found that around 2/3 of the patients who were taking antipsychotics were male and the mean age was 33.8 ± 10.2 years (Ouanes et al., 2020). Prescription of antipsychotics were observed to be more for male than for female in other parts of the world as well (Ramadas et al., 2010). One of the main reasons for the higher number of female patients in our study was due to the inclusion of all diagnoses from the general hospitals for which antipsychotics were prescribed. This exaggerated the numbers due to the large number of prescriptions of prochlorperazine for treating non-mental health diseases, such as BPPV, IBS, vomiting and migraine, many of which are more prevalent in women. When the number of patients from the MHH alone was considered, our study also found that men were prescribed more antipsychotics than women, as reported in other studies.

Prochlorperazine was the most prescribed antipsychotic in the general hospitals, followed by quetiapine and flupentixol. This pattern was slightly different in the MHH as second-generation antipsychotics (SGAs) were the most prescribed with quetiapine as the first, followed by olanzapine and risperidone. This pattern was much similar to studies performed in the Middle East previously where the most frequently prescribed antipsychotics were risperidone, olanzapine and quetiapine (Alkhadhari et al., 2015). A study in the United Kingdom also found that the most prescribed antipsychotics were SGAs, particularly quetiapine with 28%, followed by olanzapine (25%) and aripiprazole (14%). This result was similar to the pattern of antipsychotic prescribing at MHH in Qatar. Another study in Sudan showed that SGAs were prescribed to 84% of the patients and 83% were taking olanzapine (Seshadri et al., 2017).

FGAs and SGAs are FDA approved mainly for the treatment of schizophrenia and bipolar disorders and recently a few SGAs have also been approved for other MHDs. Since it was introduced, the prescribing of the SGAs has increased rapidly in different settings and expanded beyond the FDA approved indications, even though their therapeutic efficacy and adverse effects in off-label uses are not well understood (Maglione et al., 2011). A review which looked at 84 published studies on the off-label use of atypical antipsychotics concluded that there was limited evidence on the efficacy of off-label indications, with a few exceptions such as ASD, for which aripiprazole has been approved. Using quetiapine and olanzapine as adjunctive therapy for major depressive disorder has also received approval. Antipsychotics, especially risperidone and aripiprazole, may be used cautiously as first-line pharmacological augmenting agents for OCD patients who are not responding to selective serotonin reuptake inhibitors (SSRI) and cognitive behavioral therapy (CBT), but at low doses and should be monitored at 4 weeks to determine the efficacy (Veale et al., 2014). Current evidence suggests that among patients augmented with antipsychotics, one in three SSRI-resistant OCD patients will show a response (Thamby and Jaisoorya, 2019). However, there is considerable evidence that SGAs can increase the chances of adverse events, including sedation, weight gain and gastrointestinal problems. For example, the discontinuation of thioridazine use was based on reported evidence of severe cardiac arrhythmias (Maher, 2011; McKean and Monasterio, 2012). Several studies reported that quetiapine, olanzapine, and risperidone are the most common antipsychotics prescribed for off-label use (Maglione et al., 2011; Seshadri et al., 2017).

In our study, the off-label use of antipsychotics represented the majority of diagnostic indications (∼69%) of all prescribed antipsychotics, ∼56% for MHDs and 89% for non-MHDs. In comparison, the off-label use of antipsychotics in the United States was 60% (Alexander et al., 2011). Many studies showed that large numbers of older adults were prescribed antipsychotic medications for an off-label use in the United States, Canada, and Europe (Piccinni et al., 2020). Prescription of antipsychotics for challenging behavior was observed frequently rather than for patients with diagnosed MHDs. As we mentioned before, these medications are associated with serious adverse effects, especially in elderly patients with dementia (Kheirbek et al., 2019).

In our study, the off-label use of prochlorperazine and flupentixol were high, followed by a combination of fluphenazine and nortriptyline, with the brand name Motival^®^. The most common off-label indications were IBS, anxiety and stress related disorders, BPPV, migraine and pain management. Psychological and social factors may affect the development of IBS (Sinagra et al., 2012). A few studies have found that antipsychotics might have a role in the treatment of IBS, even though there is no clear evidence about their efficacy and safety (Pae et al., 2013).

Antipsychotics or dopamine antagonist antiemetics have been used off-label as second-line treatments for patients with migraine who do not tolerate triptans. Prochlorperazine can be an effective treatment for patients with acute as well as chronic headache (Mayans and Walling, 2018). In our study, prochlorperazine was the main antipsychotic used for migraine, in 94% of the patients. Also, antipsychotics were used in Qatar for patients with chronic pain. However, there is not enough evidence to show the clinical effectiveness of antipsychotics in pain management. A systematic review in chronic pain management reported that olanzapine, quetiapine, risperidone and aripiprazole were the most common off-label antipsychotics used for pain management. Among these SGAs, olanzapine demonstrated consistent evidence for its efficacy, while quetiapine did not show efficacy in pain management (Jimenez et al., 2018).

Furthermore, one of the common off-label uses of antipsychotics was for anxiety disorder. FDA has not approved any SGAs for this indication except trifluoperazine, and this drug is not prescribed widely due to safety concerns in the population. In Qatar, flupentixol and quetiapine are the most commonly used antipsychotics for treating patients with anxiety. One of the largest randomized controlled trials with quetiapine, paroxetine, and placebo, showed a similar rate of remission for both a higher dose of quetiapine and paroxetine compared with placebo (Baune, 2008).

Off-label prescribing of FGAs did occur in the past but was limited due to their extrapyramidal syndrome (EPS) adverse effects. The introduction of SGAs, which have less EPS, has led to an increase in their use significantly. From the mid-1990s to the 2000s, different studies in several countries had shown a significant increase around 2- to 5-fold in SGA prescribing. Moreover, in 2010 the global sales of antipsychotics were US$25.4 billion, which was the seventh biggest pharmacological classification group and quetiapine was the fifth biggest selling medication. In the United States, the cost burden for off-label use was approximately US$5 billion of the US$13 billion spent on antipsychotics in 2007 (McKean and Monasterio, 2012).

Wiechers et al., highlighted some possible reasons for such clinical practice of prescribing antidepressant and/or antipsychotic medications in the absence of a MHD diagnosis. First, it may be due to the concern about stigma associated with an indication of MHD in the general medical settings (Wiechers et al., 2013). Second, in the general medical setting, a potentially strong emphasis on symptom-based treatment is possible rather than a diagnosis-based treatment. We have also observed this in our study that some of the recorded indications such as delirium, hallucination, feeling sad and abnormal behavior have noted psychiatric symptoms with prescribing of medications in the absence of psychiatric diagnosis. The main prescribers for several of the psychotropic medications were from general medicine clinics rather than from the psychiatry department. Third, it might be due to patient or caregiver demand not to record MHDs in their file. Another reason may be the continuation of psychotropic drug prescriptions even when clinical indications are no longer present. Finally, poor documentation of diagnostic indications for prescribing psychotropic medications could be one of the reasons as well (Wiechers et al., 2013).

It is important to clarify that all the off-label use of antipsychotics are not without evidence and inappropriate. In some cases, as we highlighted earlier, it is supported by some of the key guidelines like NICE or there is evidence from several studies for the clinical effectiveness of this non-approved FDA indication. Sometimes such off-label use is a part of the process of expanding the approval for some indications, as medications are used off-label from time to time to treat disorders for which they are eventually approved. In other cases, unlicensed use maybe due to limited therapeutic options that are available to treat specific illness. A favorable benefit-risk ratio in this case can often justify the off-label use. In summary, physicians have the right to use medications for off-label use indications as it is their responsibility to provide the best possible care for the patients. Nevertheless, this decision should be careful and justified and supported by evidence. Also, it is important to explain to the patients about the unlicensed use of these medications and document this in the system. For example, if antipsychotics were prescribed for IBS or migraine, the doctor should explain why these medications were prescribed and what benefits and adverse effects might be expected. The consequences of unlicensed use of antipsychotics should be monitored carefully and any benefits routinely checked and recorded (Stroup and Gray, 2018).

To the best of our knowledge, this is the first comprehensive study on prescribing pattern of antipsychotics in the Middle East to examine the off-label use of antipsychotics. The data was extracted from the largest healthcare providers in Qatar including the primary care and the mental health hospital, which is leading the psychiatric services in the country, and hence covering most of the Qatari patients who are taking antipsychotics. One of the limitations of the study is that we were relying purely on EMR documentation. The diagnoses entered may not be accurate or updated as it depends on the vigilance of the physician entering the diagnosis in a busy clinical practice. We did not analyse other off-label aspects such as using these medications outside the licensed dose range and whether the patients tried any psychotropic medications before or changed from psychotropic to another medication due to adverse effects or poor response. We also did not collect data on adherence, persistence and polypharmacy, which may influence the prescription pattern and off-label prescriptions. However, polypharmacy (Ouanes et al., 2020) and treatment non-compliance (Kassis et al., 2014) were reported from Qatar in recent studies.

## Conclusion

Here we have reported population-level prescription trends of antipsychotics, revealing the off-label and non-MHD use of several medications. Based on these findings, we would advocate educating prescribers from non-mental health departments about adhering to evidence and guidelines to ensure patient safety when prescribing antipsychotics. The prescribing pattern also provides information on which drugs should be prioritized for further in-depth studies, for example, in understanding the rate of adverse effects of these medications in the population. Furthermore, reasons for the inter-individual variability of response to these medications should be investigated, especially in terms of how genetic variants may lead to such variability. Integration of the prescription data described here with pharmacogenomic, administrative and clinical data (e.g., hospitalization, results from diagnostic examinations, outcome etc.), will help in determining the course of action for implementing personalized and precision medicine in the country and beyond.

## Data Availability

The datasets presented in this study can be found in online repositories. The names of the repository/repositories and accession number(s) can be found in the article/Supplementary Material.

## Abbreviations

ASD, autism spectrum disorders; ATC, Anatomical Therapeutic Chemical classification; ADHD, attention-deficit hyperactivity disorder; BPPV, benign paroxysmal positional vertigo; CBT, cognitive behavioral therapy; EPS, extrapyramidal symptom; FDA, U.S. Food and Drug Administration; FGAs, first-generation antipsychotics; HMC, Hamad Medical Corporation; IBS, irritable bowel syndrome; MDD, major depressive disorder; MHDs, mental health disorders; MHH, mental health hospital; MRC, Medical Research Centre; NICE, National Institute for Health and Care Excellence; OCD, obsessive compulsive disorder; PHCC, Primary Healthcare Corporation; PTSD, post-traumatic stress disorder; SGAs, second-generation antipsychotics; SSRI, selective serotonin reuptake inhibitors.
